# Enhancing the Chinese writing skills of non-Chinese speaking students

**DOI:** 10.1186/2193-1801-4-S2-O1

**Published:** 2015-11-27

**Authors:** Joanna Kar-wai Cheng

**Affiliations:** Research Support Unit, Vocational Training Council, Hong Kong

## Background

A majority of the ethnic minority students living in Hong Kong acknowledge the importance of knowing the Chinese language for their future education and employment opportunities in Hong Kong. However, a survey revealed that 91% of respondents did not know how to write Chinese or considered their writing skills to be poor [1]. Another survey [2] found that 31% of those who had lived in Hong Kong for over 10 years still found the language barrier to be the greatest obstacle to finding a job. Despite acquiring basic spoken Cantonese, written Chinese was still difficult for them. Both reading and writing skills are often required for employment, but these skills are considered difficult to acquire in the current education system [3]. This study aimed to enhance the skills of non-Chinese speaking students (NCS), largely ethnic minority students, in writing Chinese.

## Methods

Action research was conducted with a teaching practitioner who taught Chinese writing, starting with learning Hanzi (Chinese characters). The stages of an action research project cycle, plan, act, observe and reflect, were followed. The first cycle addressed the question of whether the use of a Chinese Alphabet Teaching System [4] can assist NCS in learning Hanzi. During the process, students were taught how to decipher Hanzi, to remember Hanzi spellings, to orally spell out each Hanzi and to type into a computer in the same way that they wrote Chinese. The learning process was observed and the teaching strategies were reviewed and enhanced. In the second cycle, the revised question was whether the students could enhance their Hanzi writing skills and become motivated to learn Chinese. A total of 48 NCS participated in the study: 8 primary and 26 secondary school students attended a 16-hour course on writing Hanzi using the Chinese alphabet. A group of 10 adults without a background in learning Chinese attended a 40-hour course. A 6-hour interest course was designed for 4 adults. Through pre-course and post-course surveys, data were collected on their interest in learning Chinese, and the perceived difficulty of writing Chinese. Unstructured interviews were conducted with the students.

## Results

The Chinese Alphabets Teaching System was effective in helping NCS to identify the detailed structure of each Hanzi. Multimodal teaching strategies assisted students to memorise the Chinese alphabet. In the pre-course survey, 83% of the 48 students rated their interest in learning Chinese from 1 to 4 on a 10-point scale. Their lack of interest was probably due to fear. However, in the post-course survey, 85% of students rated their interest from 7 to 10. Similarly, before the training, 97% of students rated the difficulty of learning Chinese from 7 to 10, which means they found it very difficult. After the training, only 10% of students still rated it between 7 and 10, whereas 68% of students rated it between 1 and 4, indicating that the majority found writing Chinese less difficult.

## Conclusions

Enhancing the Hanzi writing skills of the NCS helped to motivate them and enabled them to learn written Chinese.
